# *Bacillus cereus* Biovar Anthracis Causing Anthrax in Sub-Saharan Africa—Chromosomal Monophyly and Broad Geographic Distribution

**DOI:** 10.1371/journal.pntd.0004923

**Published:** 2016-09-08

**Authors:** Kym S. Antonation, Kim Grützmacher, Susann Dupke, Philip Mabon, Fee Zimmermann, Felix Lankester, Tianna Peller, Anna Feistner, Angelique Todd, Ilka Herbinger, Hélène M. de Nys, Jean-Jacques Muyembe-Tamfun, Stomy Karhemere, Roman M. Wittig, Emmanuel Couacy-Hymann, Roland Grunow, Sébastien Calvignac-Spencer, Cindi R. Corbett, Silke R. Klee, Fabian H. Leendertz

**Affiliations:** 1 National Microbiology Laboratory, Public Health Agency of Canada, Winnipeg, Manitoba, Canada; 2 Project group Epidemiology of Highly Pathogenic Microorganisms, Robert Koch-Institute, Berlin, Germany; 3 World Wide Fund for Nature - Germany, Berlin, Germany; 4 Center for Biological Threats and Special Pathogens, Robert Koch-Institute, Berlin, Germany; 5 Paul G. Allen School for Global Animal Health, Washington State University, Pullman, Washington, United States of America; 6 World Wide Fund for Nature - CAR, Bangui, Central African Republic; 7 Institut National de Recherche Biomédicale, Kinshasa, Congo (DRC); 8 Max-Planck-Institute for Evolutionary Anthropology, Department of Primatology, Leipzig, Germany; 9 Taï Chimpanzee Project, Centre Suisse de Recherches Scientifiques, Abidjan, Côte d’Ivoire; 10 Laboratoire National d’Appui au Développement Agricole, Laboratoire Central Vétérinaire de Bingerville, Côte d’Ivoire; 11 Department of Medical Microbiology, University of Manitoba, Manitoba, Canada; University of Tennessee, UNITED STATES

## Abstract

Through full genome analyses of four atypical *Bacillus cereus* isolates, designated *B*. *cereus* biovar anthracis, we describe a distinct clade within the *B*. *cereus* group that presents with anthrax-like disease, carrying virulence plasmids similar to those of classic *Bacillus anthracis*. We have isolated members of this clade from different mammals (wild chimpanzees, gorillas, an elephant and goats) in West and Central Africa (Côte d’Ivoire, Cameroon, Central African Republic and Democratic Republic of Congo). The isolates shared several phenotypic features of both *B*. *anthracis* and *B*. *cereus*, but differed amongst each other in motility and their resistance or sensitivity to penicillin. They all possessed the same mutation in the regulator gene *plcR*, different from the one found in *B*. *anthracis*, and in addition, carry genes which enable them to produce a second capsule composed of hyaluronic acid. Our findings show the existence of a discrete clade of the *B*. *cereus* group capable of causing anthrax-like disease, found in areas of high biodiversity, which are possibly also the origin of the worldwide distributed *B*. *anthracis*. Establishing the impact of these pathogenic bacteria on threatened wildlife species will require systematic investigation. Furthermore, the consumption of wildlife found dead by the local population and presence in a domestic animal reveal potential sources of exposure to humans.

## Author Summary

Anthrax has historically been attributed to a single cluster within the *Bacillus cereus* complex denoted as *B*. *anthracis*. Here, we demonstrate the existence of a distinct clade of *B*. *cereus* isolates causing anthrax-like disease in a range of wild and domestic mammals in tropical Africa. These strains, designated *B*. *cereus* biovar anthracis, combine bacteriological and molecular features of *B*. *cereus* and *B*. *anthracis*. Many questions about the epidemiology, biology and impact of this cluster of anthrax causing *B*. *cereus* still remain open. On the technical side it will be important to adapt diagnostic methods for the detection of such atypical *B*. *cereus* strains–through the inclusion of molecular tools for the detection of the *B*. *anthracis* virulence plasmids that appear to be the prerequisite to cause disease. Through reliable detection of a broad range of *B*. *cereus* group isolates causing anthrax-like disease it will be possible to assess the distribution and diversity of these pathogens and their impact on public health and wildlife populations.

## Introduction

*Bacillus anthracis* has been classically defined as a clade with low genomic diversity within the *B*. *cereus sensu lato* group, whose members carry two virulence plasmids, pXO1 and pXO2, and exhibit a set of known phenotypic characteristics [1–3]. Found in many parts of the world, the organism still causes significant losses in domestic and wild animal populations and sometimes fatal infections in humans [4]. However, cases of anthrax-like disease caused by non *B*. *anthracis* members of the *B*. *cereus* group have also been identified, affecting both animal and human populations [5–8]. Early descriptions involved fatal anthrax-like infections in wild western chimpanzees (*Pan troglodytes verus*) in Côte d’Ivoire in 2001 and 2002 followed by wild central chimpanzees (*P*. *t*. *troglodytes*) and a western lowland gorilla (*Gorilla gorilla gorilla*) in Cameroon in 2004 and 2006 [9–11]. Strains isolated have been shown to carry plasmids almost identical to pXO1 and pXO2 (designated pCI-XO1 or pBCXO1 and pCI-XO2 or pBCXO2, respectively, in previous publications [12, 13]). As these atypical *B*. *cereus* types share various properties but differ significantly from *B*. *anthracis* [14], they were aptly named *B*. *cereus* biovar (bv) *anthracis*. On phenotypic level, the strains combined features of *B*. *anthracis* (lack of haemolyis and phospholipase C activity) and *B*. *cereus* (resistance to the diagnostic gamma phage, motility). Like *B*. *anthracis*, the strains from Côte d’Ivoire were sensitive to penicillin, but the strains from Cameroon were resistant [14]. Multi-locus sequence typing [15] showed the same sequence type for all *B*. *cereus* bv anthracis strains. Whole genome sequencing of one isolate from Côte d’Ivoire revealed the presence of six genomic islands (12–22 kb in size) and a small, 14 kb plasmid (pCI-14) with unknown function [12]. With a few exceptions, the sequences of these islands were only detected in *B*. *cereus* bv anthracis, whereas Island III, a putative prophage, was distributed among further strains of the *B*. *cereus* group [12]. Island VI was absent from the Cameroon isolates, and pCI-14 was only detected in some isolates from Côte d’Ivoire. The pleiotropic regulator PlcR, known to control expression of multiple genes including those related to virulence within the *B*. *cereus* group [16] is inactive both in *B*. *anthracis* due to a nonsense mutation [17] and in *B*. *cereus* bv anthracis due to a frameshift mutation which results in an altered C-terminus of the protein [12]. To some degree, virulence and virulence gene regulation are similar in *B*. *cereus* bv anthracis and classic *B*. *anthracis*. Small animal models indicate a similar level of virulence (assessed by determination of lethal doses) after infection with wild type strains, and regulation of the toxin and capsule genes by the global regulator AtxA was shown [13]. Differences in virulence account for the fact that besides the typical polyglutamate capsule of *B*. *anthracis*, an additional capsule composed of the polysaccharide hyaluronic acid was detected in *B*. *cereus* bv anthracis. This capsule, which is encoded by the *hasACB* operon on plasmid pXO1, is lacking in *B*. *anthracis* due to a mutation in the *hasA* gene [13].

Here we report the isolation, characterization and genome sequencing of additional atypical *B*. *cereus* group members isolated from wild and domestic animals sampled in Cameroon (hereafter referred to as CAM strain), the Central African Republic (RCA strains) and the Democratic Republic of Congo (DRC strain). Phylogenomic analyses provide evidence that these strains and those from Côte d’Ivoire (CI strains) belong to a single chromosomal clade clearly distinct of the *B*. *anthracis* clade.

## Methods

### Sampling and cases

**Fig 1:**
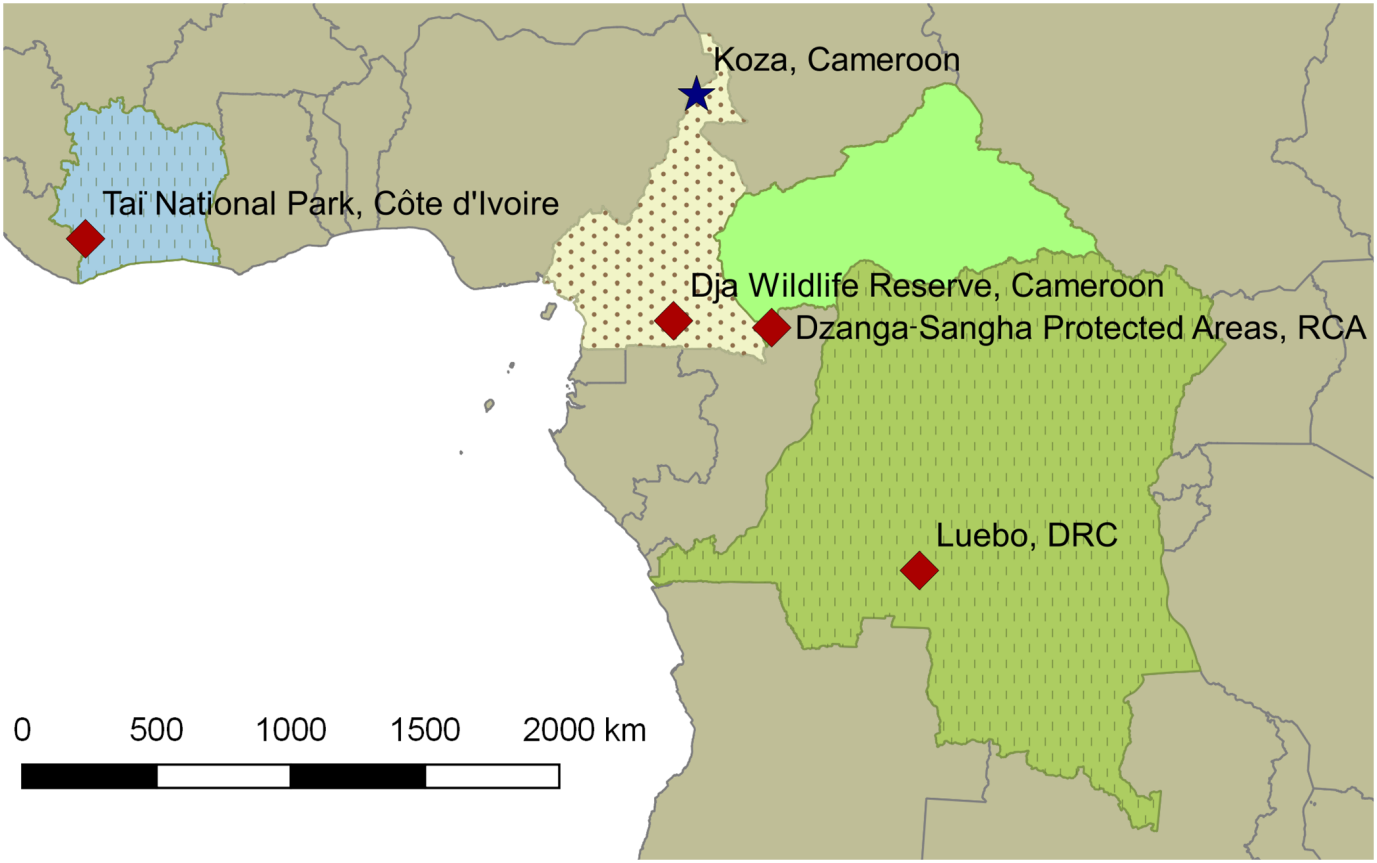
Geographic locations of carcasses. Red diamonds show sites where *Bacillus cereus* bv anthracis isolates have been detected, the star represents the site where the bovine isolate *B*. *cereus* JF3964 was found. Locations (GPS data) and animal carcasses for *B*. *cereus* bv anthracis are: Taï National Park (CI)–chimpanzee (5°50.876’N, 7°19.679’W); Dja Wildlife Reserve (CAM)–gorilla, chimpanzee (3°07.589’N, 13°06.543’E); Dzanga-Sangha (RCA)–gorilla, chimpanzee, elephant (2°53.747’N, 16°24.208’E); Luebo (DRC)–goat (5°21.074’S, 21°25.298’E). Strain JF3964: Koza (CAM)–cattle (10°57.769’N, 13°55.560’E).

All sampling sites are indicated in Fig 1.

In March 2012, 40 blood and tissue samples were collected from livestock (goats (n = 9), pigs (n = 23) and sheep (n = 1)) in Luebo, a forested town within the Kasai district of the Democratic Republic of Congo (DRC). Among the animals sampled, one goat had recently died while another was described by residents as being sick. The samples were tested on-site during a joint training project led by the Public Health Agency of Canada and the Institut National de Recherche Biomédicale, DRC.

In September 2012, eco-guards discovered a forest elephant (*Loxodonta cyclotis*) carcass in the Dzanga-Ndoki National Park, part of the Dzanga Sangha Protected Areas (DSPA) complex, in the Central African Republic (RCA, approximately 1500 miles away from DRC). The carcass was still intact and no signs of poaching were visible. A necropsy was performed and samples taken the following day in the course of a joint World Wide Fund for Nature / Robert Koch-Institute mission to investigate causes of death amongst wildlife in the area using full personal protective equipment [11] due to a history of highly pathogenic microorganisms in the area and species affected [18]. At this point the carcass had been partly opened, presumably by scavengers or humans. Five days later, an ape carcass (later confirmed by genetic analyses as a central chimpanzee) was discovered in a tree nest in the same area. Bone and skin samples were collected from the carcass, which was in an advanced stage of decomposition. Finally, in January 2013, a western lowland gorilla was found dead in the same area. The three-year-old male was part of a closely monitored group habituated to humans, and had not shown any signs of illness the previous day. Since no veterinarian was on site at this point, only deep nasal swabs were taken from the carcass by specifically instructed and protected biologists. Samples were preserved in tubes with and without preservative RNAlater (Ambion/Life Technologies, Darmstadt, Germany). All three RCA carcasses were found within a radius of five kilometres.

### Ethics statement

Samples have not been collected in the course of research projects (and therefore no permit numbers exist), they have been collected on request and as part of collaboration between the field site and the according wildlife authority of RCA (*Ministère d’Eaux et Fôret*, *Chasse et Peche* and *the Ministère de l’Education Nationale*, *de l’Alphabetisation*, *de l’Enseignement Superieur*, *et de la Recherche*) to investigate causes of death in wildlife of the region. The finding of the carcasses and later of according results of analyses have been communicated immediately to the authorities and been used to warn the local population. Samples from domestic animals from DRC have been collected in the course of collaboration with the INRB, no special permission for such sampling is required.

All wildlife samples have been exported under permission of the according CITES (Convention on International Trade in Endangered Species of Wild Fauna and Flora). The local veterinary authorities of DRC and RCA, provided a certificate of origin as requested by the German veterinary authorities (*Senatsverwaltung für Justiz und Verbraucherschutz Abteilung V—Verbraucherschutz Referat V A—Lebensmittel- und Veterinärwesen*, *Gentechnik Stellenzeichen—V A VET 0*.*2*, Berlin, Germany) for import of samples.

### Initial pathogen detection

DNA of the RCA samples was extracted from various tissues following the protocol of the NucleoSpin RNA II Kit (Macherey-Nagel GmbH & Co. KG, Düren, Germany) using the NucleoSpin RNA/DNA Buffer Set for parallel purification of genomic DNA, excluding step 7 for DNA digestion. DNA was prepared on separate days and samples were treated from one animal at a time. To test for the presence of *B*. *cereus* bv anthracis real-time PCR assays were performed targeting the *pagA* gene for pXO1 [19], the *capB* gene for pXO2, and a marker specific for genomic island IV of *B*. *cereus* bv anthracis [12]. The primers and TaqMan-probes for capB and IslandIV were designed using the Primer Express V2.0 software (Applied Biosystems, Darmstadt, Germany) and ordered from Metabion (Martinsried, Germany). Primer and probe sequences are shown in S3 Table. Real-time PCR conditions were applied as described before [19].

Genetic identification of the great ape skin samples was performed (by tissue extraction as above, followed) by a pan-mammal assay as described by Calvignac-Spencer et al. 2013 [20].

Blood samples from two goats from DRC were tested for *B*. *anthracis* using real-time PCR screening in the field. The field testing comprised of DNA extraction via use of the Qiagen ViralAmp (Qiagen, Hilden, Germany) kit as per manufacturer’s directions, followed by real-time PCR using an assay targeting both the pXO1 and pXO2 plasmids (in-house developed) in addition to a chromosomal region with the gyrase gene [21].

### Bacterial cultures

Liver samples (conserved in RNAlater) and untreated fat tissue of the elephant from RCA were cut into small pieces and used for cultivation on different selective (blood trimethoprim agar, Cereus Ident agar, Cereus selective agar) and non-selective (sheep blood agar) media [14]. To test for the presence of spores, a small piece of each tissue was additionally placed into 500 μl of saline and vegetative bacteria were inactivated by heating at 65°C for 30 min. The nasal swab (also conserved in RNAlater) taken from the gorilla in RCA was transferred into 900 μl saline and one half was directly spread on different agar plates and the remaining half was heat inactivated. After heat treatment, tissue material and supernatant was spread on different media as describe above. Heat-inactivated blood from the DRC was also cultured for the presence of *Bacillus*-like organisms on non-selective blood agar.

Any colonies suspicious for *B*. *cereus* bv anthracis were dispensed in water, heat inactivated at 95°C for 30 min and used directly for real-time PCR assays as described above. If the presence of *B*. *cereus* bv anthracis was confirmed, the corresponding bacterial isolates were tested for motility by growth on a semi-solid tryptic soy agar (0.3% agar) as well as for susceptibility to penicillin G and the diagnostic gamma phage as described before [14].

### Molecular analysis of bacterial isolates

DNA was extracted from bacterial isolates using the DNeasy Blood & Tissue Kit (Qiagen) for the RCA isolates and the Epicentre MasterPure kit (Madison, Wisconsin, USA) for the DRC isolate. DNA was tested for the presence of genomic islands I to VI and plasmid pCI-14 using primers targeting appropriate genes. Primer sequences are listed in S3 Table. Gene fragments were amplified by PCR under the same conditions as described before [12].

For whole genome sequencing, DNA from *B*. *cereus* bv anthracis CI (same isolate as sequenced previously using the Sanger method, [12]), CAM (isolate from chimpanzee), RCA (isolates from elephant and gorilla) and DRC was processed using the Nextera DNA Library Preparation Kits (Illumina, Munich, Germany) as per manufacturers’ instructions. Miseq reagent kits with v2 chemistry (500 cycle) were used on the Miseq platform (Illumina) to generate sequence data and fed through an in-house bioinformatic pipeline described below.

MLST [15] was performed to confirm that also this method would be capable of differentiating *B*. *cereus* bv anthracis from *B*. *anthracis* and we investigated the existence of the frameshift mutation in the *plcR* gene and the integrity of the *hasA* gene by PCR and sequencing using standard methods.

### Bioinformatics

Strains were assembled with Spades version 2.5.1 with recommended parameters for 2 x 250 bp Illumina reads. Contigs were filtered against lengths < 200 bp [22]. Average Nucleotide identity (ANI) was determined with Jspecies blast option [23] using default parameters based on spades contig assemblies and refSeq records from NCBI (S4 Table). Canonical SNPs were confirmed manually by visual inspection with Tablet from generated pileups and alignment with samtools and SMALT respectively [24, 25] (http://www.sanger.ac.uk/resources/software/smalt/; version 0.7.5).

### Phylogenetic analyses

Core pipeline analyses were generated with an in-house pipeline available at github (https://github.com/apetkau/core-phylogenomics; commit version 0317413ba9). To ensure consistent data across all strains, in silico error free illumina reads were generated using Wombac from contigs available on NCBI. This was only done for strains without any publicly available raw reads http://www.vicbioinformatics.com/software.wombac.shtml (version dated Oct 3, 2013). All public accessible *Bacillus* strain sequences on NCBI (S5 Table) were downloaded on June 25, 2015 and used in an initial round of phylogenetic analyses for each reference. An iterative approach was used to exclude strains based on their core percentage to the reference strain *B*. *anthracis* Ames Ancestor. Strains with less than 50% homology were removed from final tree(s) analyses. Core pipeline criteria for high quality SNPs (hqSNPs) were minimum base pair and mapping quality > = 30 phred score with 25 read coverage with 75% of consensus. SNPs were concatenated together to create multiple meta-alignments; one for each plasmid and chromosome. Model selection was performed in a maximum likelihood framework using jModelTest v2.1.3 [26]. Phylogenetic analyses were performed in PhyML using the GTR model; branch support was estimated with Shimodaira-Hasegawa-like approximate likelihood ratio tests [27]. ML trees were rooted with TempEst v1.5 [28]. Homoplasy indices were calculated using Paup * version 4.0 [29]. Figures were generated using FigTree v1.4.1 (http://tree.bio.ed.ac.uk/software/figtree/).

## Results

### Testing for and culture of *B*. *cereus* biovar *anthracis*

DNA extracted from tissues, bones, nasal swabs and blood of the elephant, chimpanzee, gorilla from RCA and livestock from DRC were tested by real-time PCR assays targeting pXO1 and pXO2. All samples from wildlife and the samples of the sick and dead goats were positive. The presence of *B*. *cereus* bv anthracis was further confirmed by a specific real-time PCR targeting Island IV [12] (Table 1).

**Table 1:** Comparison of selected microbiological and molecular features of *Bacillus cereus* bv anthracis strains.

Feature	Strain designation (host)				
	CI (chimpanzee)	CAM (gorilla, chimpanzee)	RCA A-363/2 (elephant)	RCA A-364/1 (gorilla)	DRC 14-0024-1 (goat)
Haemolysis	No	No	No	No	No
Phospholipase C activities (PI-specific / PC-specific)	No / Weak	No / Weak	No / Weak	No / Weak	No / Weak
Gamma phage	Resistant	Resistant	Resistant	Resistant	Resistant
Penicillin G	Sensitive	Resistant	Resistant	Resistant	Resistant
Motility	Yes	Yes	Yes	Yes	No
Plasmid pXO1	Present	Present	Present	Present	Present
Plasmid pXO2	Present	Present	Present	Present	Present
Genomic Islands I-V	Present	Present	Present	Present	Present
Genomic Island VI	Present	Absent	Absent	Absent	Absent
Plasmid pCI-14	Present^a^	Absent	Absent	Absent	Absent
Frameshift mutation in *plcR* gene	Present	Present	Present	Present	Present
MLST (allele numbers)^b^	34/1/83/1/18/29/5	34/1/83/1/18/29/5	34/1/83/1/18/29/5	34/1/83/1/18/29/5	34/1/83/1/18/29/5

PI, phosphatidylinositol; PC, phosphatidylcholine

^a^The plasmid is not present in all CI strains.

^b^Order of allele fragments: glpF/gmk/ilvD/pta/pur/pycA/tpi

Three new bacterial colony isolates with a typical *B*. *cereus* bv anthracis morphology were cultured from fat tissue of the elephant (specimen number A-363/2), the nasal swab of the gorilla (specimen number A-364/1) and blood of the dead goat (specimen number 14-0024-1). The presence of spores in these samples was confirmed by cultivation of bacteria after heat treatment of clinical material at 65°C for 30 min. No culture was obtained from the bones and skin of the chimpanzee as well as from any tissue from the other goat.

### Bacteriological and molecular properties

The bacteriological properties of the three new isolates resembled those described previously for the CI and CAM strains (Table 1). Like *B*. *anthracis*, the isolates were non-haemolytic, lacked the phosphatidylinositol-specific phospholipase C activity and showed a weak lecithinase (phosphatidylcholine-specific phospholipase C) activity. Unlike *B*. *anthracis*, the isolates were resistant to the gamma-phage [14]. Most colonies also showed a strong mucoid appearance, indicating the presence of encapsulated bacteria. In addition, like the CAM strain, the three new isolates were resistant to penicillin G. Like the CI and CAM strains, the forest elephant and the gorilla isolates from RCA both exhibited motility, whilst the goat isolate from DRC was non-motile. Molecular analyses were performed on bacterial DNA that was extracted from suspicious colonies isolated from the RCA and DRC samples. The presence of six genomic islands and the small plasmid pCI-14 was examined by PCR [12]. Isolates from RCA and DRC harboured genomic islands I to V, but like the CAM strain lacked genomic island VI and plasmid pCI-14 (Table 1).

Analyses of key functional genes revealed that all strains shared the frameshift mutation in the *plcR* regulator gene already described for the CI strain [12]. In addition, and unlike *B*. *anthracis*, they possessed a functional *hasACB* operon on pXO1 allowing synthesis of a second capsule type composed of hyaluronic acid [13, 30]. Interestingly, from the whole genomic sequencing data (see below) a pre-mature stop codon was detected in one gene (*fliP*) of the flagella gene cluster of the DRC strain, thereby explaining the lack of motility observed for this strain. Isolate characteristics are summarized in Table 1.

### Phylogenomic analysis

We sequenced the genomes of the three new isolates (RCA elephant and gorilla, DRC), concurrently with the CAM and CI strains and compared their chromosomal and plasmid sequences (unclosed) to a collection of complete genomes of *B*. *cereus*, *B*. *thuringiensis* and *B*. *anthracis*. Common genes distributed across the chromosomes of all genomes analysed represent the chromosomal core, and altogether, 3,091,761 core coding positions were detected. Phylogenomic analyses performed in a maximum likelihood (ML) framework using variable coding positions (single nucleotide polymorphisms; SNPs) unambiguously identified 169,492 positions in all genomes for chromosomal sequences. Similar analyses were performed to identify the core regions of the two plasmids of *B*. *anthracis*, *B*. *cereus* bv anthracis and a few other members of the *B*. *cereus* group which also possess pXO1. There were 697 and 250 variable positions determined for pXO1 and pXO2 plasmid sequences, respectively (most members of the *B*. *cereus* group do not possess these plasmids). Despite the reduced size of these data sets, unique SNPs were observed for all strains.

**Fig 2:**
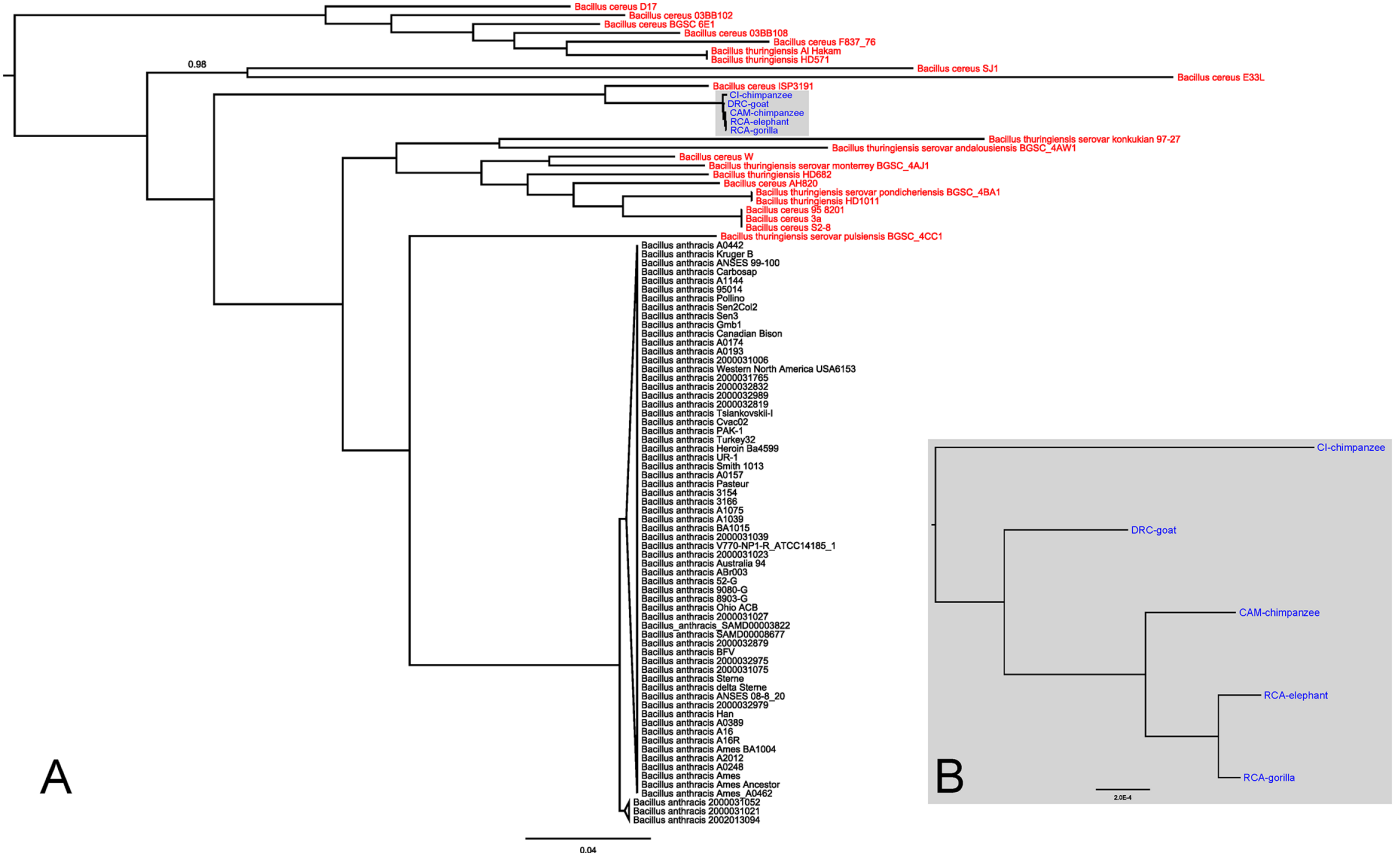
Maximum likelihood tree based on core chromosomal SNP data. A: Full tree. B: Zoom-in focused on the *Bacillus cereus* biovar anthracis clade. *Bacillus anthracis* sequences are black, *Bacillus cereus* and *Bacillus thuringiensis* red and *B*. *cereus* bv anthracis blue. Branch support values were estimated by approximate likelihood ratio tests and are only reported for these internal branches not supported by maximal values. This tree was rooted with TempEst v1.5.

Maximum likelihood trees derived from the analysis of chromosomal sequences strongly supported the existence of a clade comprising all *B*. *cereus* bv anthracis strains (Fig 2A). Of note, this clade was not closely associated to the *B*. *anthracis* clade. Instead, the closest relative of *B*. *cereus* bv anthracis appeared to be another member of the *B*. *cereus* group, i.e. *B*. *cereus* ISP3191 [31, 32], and, conversely, a number of *B*. *cereus* and *B*. *thuringiensis* strains appeared more closely related to *B*. *anthracis* than *B*. *cereus* bv anthracis. *B*. *cereus* bv anthracis strains did not differ amongst each other at the chromosome level at more than 450 positions (~0.01% difference), which was markedly lower than the maximum ~1,000 SNPs observed amongst *B*. *anthracis* chromosomes (~0.04%). However, the number of SNPs among *B*. *cereus* bv anthracis strains will probably increase with larger sampling numbers. Furthermore, approximately 39,000 SNPs separated isolates within the *B*. *cereus* bv anthracis and *B*. *anthracis* clades; in comparison, *B*. *cereus* ISP3191 was only separated from *B*. *cereus* bv anthracis by an average of ~12,000 SNPs. Within the *B*. *cereus* bv anthracis clade, isolates from Central Africa (RCA and CAM) were more closely related to each other than to the DRC and CI isolates (Fig 2B).

**Fig 3:**
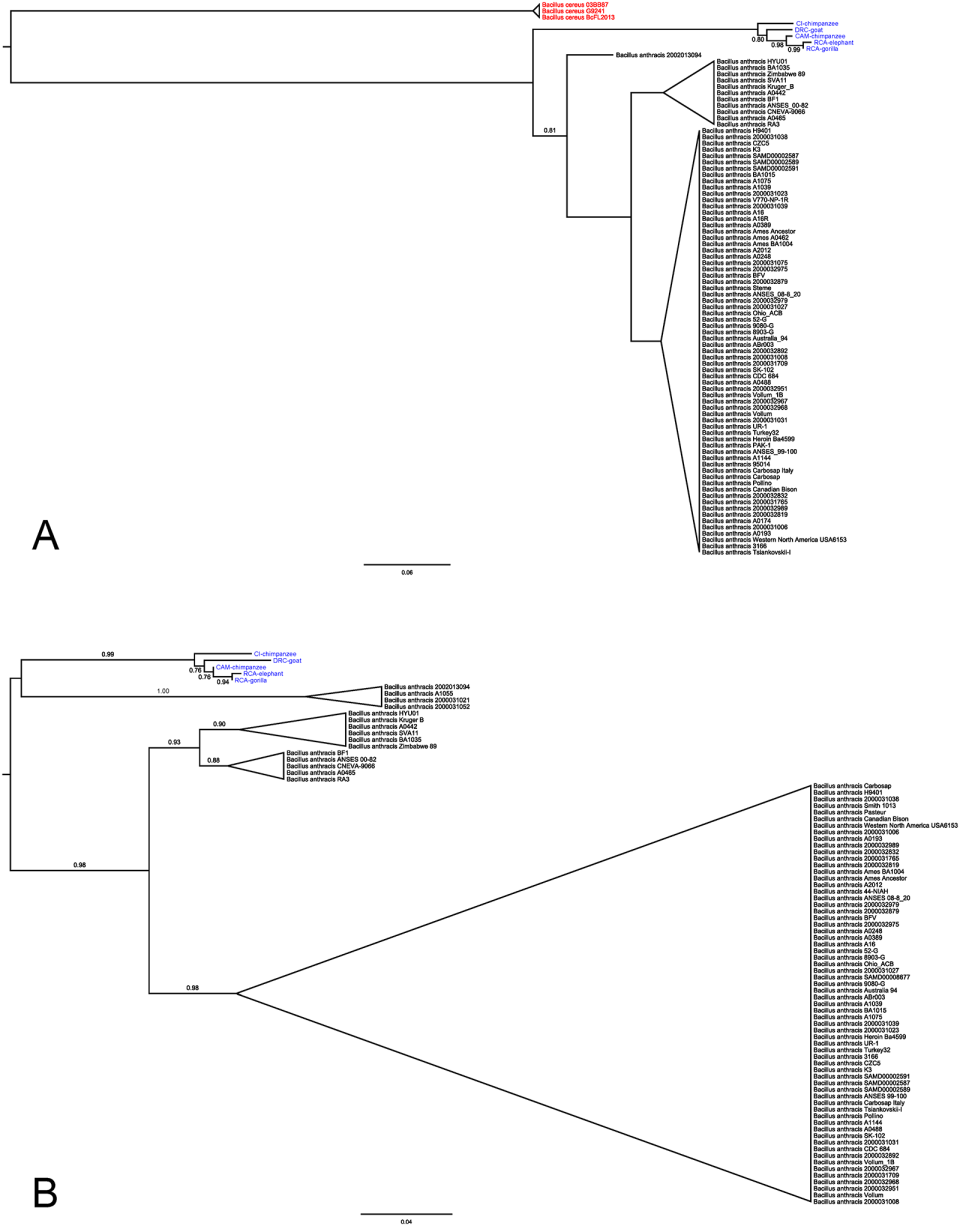
Maximum likelihood tree based on core plasmid SNP data. A: pXO1. B: pXO2. *Bacillus anthracis* sequences are black, *Bacillus cereus* red and *Bacillus cereus* biovar anthracis blue. Branch support values were estimated by approximate likelihood ratio tests and are only reported for these internal branches not supported by maximal values. The trees were rooted with TempEst v1.5.

ML trees derived from pXO1 and pXO2 data sets also supported the monophyly of *B*. *cereus* bv anthracis isolates (Fig 3). *B*. *anthracis* isolates also formed a clade using sequence data from pXO1, but not pXO2. In contrast, *B*. *anthracis* strains A1055, 2000031052, 2000031021 and 2002013094 belonging to the rare C lineage [3] appeared more closely related to *B*. *cereus* bv anthracis isolates than to other *B*. *anthracis* isolates. This pattern, however, entirely depends on the placement of the root which, in the absence of an appropriate outgroup, was determined with TempEst by minimizing the variance of root-to-tip distances (i.e. assuming the tree mostly behaves in a clocklike manner [28]). This observation should therefore be regarded as preliminary and certainly deserves further investigation. For both plasmids, the low number of polymorphic sites prevented the full determination of the branching order within *B*. *cereus* bv anthracis. The clade comprising CAM and RCA isolates was, however, satisfactorily resolved. The lack of support for the second deepest node in the clade did not allow addressing the question of the branching order of CI, DRC and CAM+RCA.

We finally determined homoplasy indices (HI) in a parsimony framework for all complete trees as well as for the *B*. *cereus* bv anthracis clade. The complete chromosomal tree was the only one to reveal significant homoplasy levels (HI = 0.47) which suggests a measurable contribution of recombination to the evolutionary history of the *Bacillus cereus* clade. In contrast, HI were very low for the complete plasmid trees (pXO1 0.01, pXO2 0.02) and for the *B*. *cereus* bv anthracis clade (chromosome 0.00, pXO1 0.00, pXO2 0.00).

To determine whether easier PCR-based methods would also identify the *B*. *cereus* bv anthracis clade, we performed multilocus sequence typing (MLST) and showed that sequences of the alleles of the seven housekeeping gene fragments for MLST [15] resulted in a sequence type, ST 935, which is unique for all isolates of *B*. *cereus* bv anthracis (Table 1). The profile has been submitted to the corresponding database (http://pubmlst.org/bcereus/).

### Accession numbers

NCBI accession numbers for sequences of *B*. *cereus* bv anthracis CI, CAM, RCA elephant (A-363/2), RCA gorilla (A-364/1) and DRC goat (14-0024-1) are from SAMN03610233 to SAMN03610237, respectively.

## Discussion

Using phylogenomic analyses, we demonstrated that several strains of *B*. *cereus* group members, designated *B*. *cereus* bv anthracis and carrying the plasmids pXO1 and pXO2, belong to a single chromosomal clade distinct from the *B*. *anthracis* clade. Importantly, we also show that members of this clade can be found throughout much of tropical Africa and can infect a variety of wildlife and domestic animals.

Strains within this clade exhibit unique genomic variation and their evolution seems to be mostly driven by mutations arising in the context of a clonal lifestyle. A putative phylogeographic pattern can be identified, which may be suggestive of continent-scale population structure and/or isolation-by-distance. This suggests that this clade has existed for quite a while and excludes the possibility of a single clone in rapid expansion. In addition, the fact that plasmid phylogenies are compatible with the same branching order seen in the chromosome phylogeny support the notion that these plasmids were only acquired once—by an ancestor of the *B*. *cereus* bv anthracis lineage—and persisted since then within this lineage.

The reason why *B*. *anthracis* is distributed worldwide and why *B*. *cereus* bv anthracis is, to our current knowledge, found rather restricted to the more humid and warm regions of tropical Africa requires further investigations but may be related to biological properties such as their capacity to sporulate under different climatic conditions. Indeed, some functional differences are observed, for example a second, pXO1-encoded capsule composed of hyaluronic acid in *B*. *cereus* bv anthracis which is an important virulence factor and is inactive in *B*. *anthracis* due to a frameshift mutation on pXO1 [13]. Interestingly, *B*. *anthracis* strain 2002013094, the only C branch strain with a pXO1 plasmid available in the NCBI database, also possesses an intact *hasA* gene. Synthesis of a hyaluronic acid capsule by this strain remains to be examined.

Unfortunately, we were not able to include in this study another known *B*. *cereus* strain harbouring pXO1 and pXO2, JF3964, isolated from cattle in Cameroon, as the genome sequence was still not determined at the time of writing [33]. However, JF3964 is lacking the chromosomal marker Ba813 that is present in CI, CAM, RCA and DRC and *B*. *anthracis* strains [14, 33], and this suggests that JF3964 does not belong to the *B*. *cereus* bv anthracis clade that we describe here. In line with this, 13 canonical SNPs [3] showed an identical pattern for the RCA, DRC, CI and CAM strains, whereas a difference of two SNPs (B.Br.002 and A/B.Br.001) was observed from the bovine isolate JF3964 (S1 Text, S2 Table). As these canSNPs were developed to identify the three main lineages constituting the *B*. *anthracis* clade, these conclusions should be considered with caution. We also performed MLVA (multiple-locus variable-number tandem repeat analysis, [34]) to compare strains of the *B*. *cereus* bv anthracis clade with isolate JF3964 and found that all strains exhibit an unique allelic profile (S1 Text, S1 Table).

Not surprisingly, on a microbiological level, the *B*. *cereus* bv anthracis strains share properties that are intermediary to *B*. *anthracis* (e. g. lack of haemolysis) and *B*. *cereus* (e. g. motility, gamma phage and penicillin resistance) [14]. The five isolates described here differ in their susceptibility to penicillin G, with the CI strain being sensitive, and the CAM, RCA and DRC strains being resistant. The only other important difference may be that the CI, CAM and RCA strains are motile, while the DRC strain is not, most likely due to a mutation in the *fliP* gene—similar to the situation in *B*. *anthracis*, where several mutations are present in the flagellar gene cluster [12].

Of interest, an environmental strain of *B*. *cereus* (ISP3191) presented itself as the closest chromosomal relative of *B*. *cereus* bv anthracis. This bacterium does not possess the *B*. *anthracis* plasmids, but does appear to comprise a plasmid with a pXO1-backbone and a plasmid with some similarity to pXO2 [32]. Ultimately, the strain could not be included in our final phylogenetic analyses of pXO2 as its inclusion resulted in the exclusion of many sites for which homology was uncertain—the core content for analysis dropped from 63% to 28%. The strain was isolated from a food source (spice) in Belgium and was sequenced as part of a large study of environmental isolates of *B*. *cereus* [31]. Interestingly, the frameshift mutation of the *B*. *cereus* bv anthracis *plcR* gene also occurs in *B*. *cereus* ISP3191 [35], whereas the genomic islands (except the relatively wide distributed Island III) are absent.

Few pathogenic *B*. *cereus* isolates are reported in the literature that do contain the elements required for pathogenicity and anthrax-like disease in humans such as G9241 [5], 03BB102 [6], 03BB87 [6], BcFL2013 [8] and Elc2 [7]. The available chromosomal and plasmid sequences of the first four isolates were distinct and did not cluster with the *B*. *cereus* bv anthracis clade. As noted by Brezillon et al. (2015), it is possible that these strains are simply the recipient host of a pXO1-like plasmid transfer [13], enabling normally non-pathogenic *B*. *cereus* isolates to cause anthrax-like disease.

Handling and consumption of wildlife and domesticated animals found dead is common practise in sub-Saharan Africa and has already led to the emergence of various infectious diseases, including the Ebola virus disease [18]. The epidemiology of *B*. *cereus* bv anthracis requires further investigation, as potential transmission pathways—deviating from what is known for *B*. *anthracis*–could present risks to public health. Furthermore, as this clade does not meet the classic diagnostic criteria for *B*. *anthracis* it may be missed by classic bacteriological methods as the causative agent of anthrax-like disease. Regardless, the descriptions and sequences obtained from this study will contribute to a better understanding of the *B*. *cereus sensu lato* group.

### Closing comment

Of note, after diagnoses of anthrax-like disease, the protected area authorities were informed and actions were put in place to monitor for further cases in DSPA. At present, no human cases have been reported in these regions.

## Supporting Information

S1 Text Further molecular characterization of isolates by MLVA and canSNP profiling. (DOCX)

S1 Table Sizes of MLVA markers in *B*. *cereus* bv anthracis strains compared to JF3964 and classic *B*. *anthracis*. (DOCX)

S2 Table Analysis of canonical SNPs in *B*. *cereus* bv anthracis strains compared to JF3964, *B*. *anthracis* C lineage and further *B*. *anthracis* lineages frequently found in Africa. (DOCX)

S3 Table Primers and TaqMan-probes for real-time PCR assays of pagA, capB and genomic island IV and primers for conventional PCRs targeting gene fragments of genomic islands I to VI and plasmid pCI-14. (DOCX)

S4 Table Reciprocal Blast Average nucleotide identity Percentage using Jspecies. (DOCX)

S5 Table Genomes used in this study. (XLSX)
