# Supplementary material for: Bacillus cereus Biovar Anthracis Causing Anthrax in Sub-Saharan Africa—Chromosomal Monophyly and Broad Geographic Distribution
Source: PLoS Negl Trop Dis. 2016 Sep 8;10(9):e0004923. doi: 10.1371/journal.pntd.0004923 (PMC5015827; doi:10.1371/journal.pntd.0004923)
Supplement: S1 Text — (DOCX) [file pntd.0004923.s001.docx]

**S1 Text.**

**Further molecular characterization of isolates by MLVA and canSNP profiling.**

Another atypical *B. cereus* isolate (JF3964) from cattle in northern Cameroon was described in 2011 (1). This strain also possessed pXO1- and pXO2-like plasmids. To allow comparison of our strains with strain JF3964, for which no genome sequence is available, we performed multiple-locus variable-number tandem repeat analysis (MLVA) (2) and single nucleotide polymorphism (SNP) typing (3).

The sizes of the eight MLVA markers were similar for the *B. cereus* bv anthracis strains and JF3964, but each strain exhibited an unique allelic profile (S1 Table). Interestingly, a deletion of one nucleotide in a C homopolymer stretch which was observed in the pXO2 MLVA locus of all *B. cereus* bv anthracis strains was also detected in strain JF3964 and in the corresponding pXO2 fragment of *B. anthracis* strains belonging to the clonal lineage C (3).

The canonical SNP profile of the atypical bovine isolate JF3964 differs in two positions from *B. cereus* bv anthracis isolates (S2 Table), in addition, JF3964 is lacking the chromosomal marker Ba813 (4) which is present in CI, CAM, RCA and DRC strains (1, 5). Therefore, this strain probably does not belong to the same clonal lineage, but represents a strain with a related chromosomal background, which also allows maintenance and replication of the *B. anthracis* virulence plasmids.

The atypical *B. cereus* strains reported here are very close in SNP profile to what has been described for the C lineage of *B. anthracis* isolates, with only one difference at A.Br.002 (3). As the canSNP framework is a pathway of acquired SNPs and the atypical isolates share the G allele at the A/B.BR.001 branch location with the ancestral C lineage, the A.Br.002 SNP would not matter in the scheme of defining their place within the phylogenetic framework. The shared allele at the A/B.BR.001 branch merely informs one that these atypical isolates are in an evolutionary state before the A and B lineages diverged from a common ancestor, which is reasonably intuitive.

**References:**

1. Pilo P, Rossano A, Bamamga H, Abdoulkadiri S, Perreten V, Frey J. Bovine *Bacillus anthracis* in Cameroon. Appl Environ Microbiol. 2011; 77(16):5818-5821.

2. Keim P, Price LB, Klevytska AM, Smith KL, Schupp JM, Okinaka R, et al. Multiple-locus variable-number tandem repeat analysis reveals genetic relationships within *Bacillus anthracis*. J Bacteriol. 2000; 182(10):2928-2936.

3. Van Ert MN, Easterday WR, Huynh LY, Okinaka RT, Hugh-Jones ME, Ravel J, et al. Global genetic population structure of *Bacillus anthracis*. PLoS One. 2007; 2:e461.

4. Patra G, Sylvestre P, Ramisse V, Therasse J, Guesdon JL. Isolation of a specific chromosomic DNA sequence of *Bacillus anthracis* and its possible use in diagnosis. FEMS Immunol Med Microbiol. 1996; 15:223-231.

5. Klee SR, Ozel M, Appel B, Boesch C, Ellerbrok H, Jacob D, et al. Characterization of *Bacillus anthracis*-like bacteria isolated from wild great apes from Cote d'Ivoire and Cameroon. J Bacteriol. 2006; 188(15): 5333-5344.
