# Supplementary material for: Bacillus cereus Biovar Anthracis Causing Anthrax in Sub-Saharan Africa—Chromosomal Monophyly and Broad Geographic Distribution
Source: PLoS Negl Trop Dis. 2016 Sep 8;10(9):e0004923. doi: 10.1371/journal.pntd.0004923 (PMC5015827; doi:10.1371/journal.pntd.0004923)
Supplement: S1 Table — (DOCX) [file pntd.0004923.s002.docx]

**S1 Table.** **Sizes of MLVA markers in *B. cereus* bv anthracis strains compared to JF3964 and classic *B. anthracis***

| Strain | MLVA marker*^a^* | | | | | | | |
| --- | --- | --- | --- | --- | --- | --- | --- | --- |
|  | vrrA | vrrB1 | vrrB2 | vrrC1 | vrrC2 | CG3 | pXO1 | pXO2 |
| CI | 290 | 271 | 162 | 511 | 820 | 158 | 120 | 134*^b^* |
| CAM | 290 | 253 | 171 | 517 | 784 | 158 | 120 | 134*^b^* |
| A-363/2 | 290 | 253 | 171 | 511 | 820 | 158 | 120 | 132*^b^* |
| A-364/1 | 290 | 253 | 171 | 511 | 820 | 158 | 120 | 132*^b^* |
| 14-0024-1 | 290 | 271 | 162 | 511 | 748 | 158 | 120 | 132*^b^* |
| JF3964 | 302 | 253 | 144 | 505 | 820 | 158 | 120 | 132*^b^* |
| Ba A1055 | 326 | 292 | 171 | 364 | 604 | 158 | absent | 132*^b^* |
| *B. anthracis* | 290-326 | 184-256 | 135-171 | 502-685 | 532-660 | 153-158 | 117-141 | 133-155 |

*^a^*Amplicon size in bp determined by PCR and sequencing

*^b^*Deletion in a C homopolymer stretch (5 x C compared to 6 x C for *B. anthracis*)
