# Supplementary material for: Bacillus cereus Biovar Anthracis Causing Anthrax in Sub-Saharan Africa—Chromosomal Monophyly and Broad Geographic Distribution
Source: PLoS Negl Trop Dis. 2016 Sep 8;10(9):e0004923. doi: 10.1371/journal.pntd.0004923 (PMC5015827; doi:10.1371/journal.pntd.0004923)
Supplement: S2 Table — (DOCX) [file pntd.0004923.s003.docx]

**S2 Table.** **Analysis of canonical SNPs in *B. cereus* bv anthracis strains compared to JF3964, *B. anthracis* C lineage and further *B. anthracis* lineages frequently found in Africa.**

| Lineage/Group | A.Br.001 | A.Br.002 | A.Br.003 | A.Br.004 | A.Br.006 | A.Br.007 | A.Br.008 | A.Br.009 | B.Br.001 | B.Br.002 | B.Br.003 | B.Br.004 | A/B.Br.001 |
| --- | --- | --- | --- | --- | --- | --- | --- | --- | --- | --- | --- | --- | --- |
| CI | T | A | A | T | C | T | T | A | T | G | G | T | G |
| CA | T | A | A | T | C | T | T | A | T | G | G | T | G |
| A-363/2 | T | A | A | T | C | T | T | A | T | G | G | T | G |
| A-364/1 | T | A | A | T | C | T | T | A | T | G | G | T | G |
| 14-0024-1 | T | A | A | T | C | T | T | A | T | G | G | T | G |
| JF3964 | T | A | A | T | C | T | T | A | T | C | G | T | A |
| C.Br.A1055 | T | G | A | T | C | T | T | A | T | G | G | T | G |
| A.Br.Vollum | T | G | A | T | A | C | T | A | T | G | G | T | A |
| A.Br.001/002 | T | A | G | C | A | T | T | A | T | G | G | T | A |
| A.Br.Aust94 | T | G | G | C | A | T | T | A | T | G | G | T | A |
| A.Br.003/004 | T | G | A | C | A | T | T | A | T | G | G | T | A |
| B.Br.001/002 | T | G | A | T | C | T | T | A | T | T | A | T | A |
