# Supplementary material for: Bacillus cereus Biovar Anthracis Causing Anthrax in Sub-Saharan Africa—Chromosomal Monophyly and Broad Geographic Distribution
Source: PLoS Negl Trop Dis. 2016 Sep 8;10(9):e0004923. doi: 10.1371/journal.pntd.0004923 (PMC5015827; doi:10.1371/journal.pntd.0004923)
Supplement: S3 Table — (DOCX) [file pntd.0004923.s004.docx]

**S3 Table.** **Primers and TaqMan®-probes for real-time PCR assays of pagA, capB and genomic island IV and
 primers for conventional PCRs targeting gene fragments of genomic islands I to VI and plasmid pCI-14.**

| Target | ORF number in NCBI database | Primer / Probe | Sequence (5’🡪3’ direction) | Amplicon size |
| --- | --- | --- | --- | --- |
| pagA | BACI_pCIXO101530 | pagA-S | CGGATCAAGTATATGGGAATATAGCAA | 204 bp |
|  |  | pagA-A | CCGGTTTAGTCGTTTCTAATGGAT |  |
|  |  | pagA-TM | FAM-CTCGAACTGGAGTGAAGTGTTACCGCAAA-TAMRA |  |
| capB | BACI_pCIXO200640 | capB-S | GGGAAAACAACTGGTACATCTGC | 144 bp |
|  |  | capB-A | AAGTGCTTCTGCTTCTAAATCAGC |  |
|  |  | capB-TM | FAM-CCTCTTTAACTACCCTGCGTTGCTCACCG-TAMRA |  |
| Genomic Island IV | BACI_c43090 | IslandIV-S | GGAGATATTAACAAGAGATGGATTGGA | 140 bp |
|  |  | IslandIV-A | CAGTAGGCTTGTCTGCTCTAATAAAATT |  |
|  |  | IslandIV-TM | FAM-ACATGCCAGCGTTTTTTGCCTCTACACA-BHQ1 |  |
| Genomic Island I | BACI_c22180 | 22180-for | GACTGTATGGTTGCTTCCAGAG | 638 bp |
| (13 kb) |  | 22180-rev | CTGTATTTCCTAAAAACTGCACC |  |
|  | BACI_c22220 | 22220-for | AGAGGACTATAAGGAGTTTATCC | 677 bp |
|  |  | 22220-rev | CAAATGCATTGATAAACTTCTTGG |  |
| Genomic Island II | BACI_c24230 | 24230-for | GTAAGAGAGGTGAATGATTACC | 535 bp |
| (12 kb) |  | 24230-rev | CTCGTATTAACTTAGGGATAGC |  |
|  | BACI_c24340 | 24340-for | GCTTTCAGTCTGTTGCCGAG | 619 bp |
|  |  | 24340-rev | AACGATGAGATTGCCACCTC |  |
| Genomic Island III | BACI_c24450 | 24450-for | GTGTATCAACTACCATCACAG | 300 bp |
| (13 kb) |  | 24450-rev | CTAGTTCCAGAAATTCTCCTC |  |
|  | BACI_c24500 | 24500-for | ATCTTCTTGCCGTAACTCGG | 438 bp |
|  |  | 24500-rev | CCTGCAATAGTTCCATTTGCC |  |
|  | BACI_c24550 | 24550-for | GTACCATGTCAGGTTGAAGG | 445 bp |
|  |  | 24550-rev | TCACCTTCTGTCAGATCTGC |  |
| Genomic Island IV | BACI_c43090 | 43090-for | CATAGGAGAAGCTTCAGAGC | 745 bp |
| (22 kb) |  | 43090-rev | CAACTCTTCCACTGTCGAAC |  |
|  | BACI_c43150 | 43150-for | CGATCAAAGGTCGATAAGGG | 748 bp |
|  |  | 43150-rev | GATTCAAGGTAGGTACTCCAG |  |
|  | BACI_c43220 | 43220-for | TTGGATTCGTTAAATGAGGAAG | 426 bp |
|  |  | 43220-rev | AAACCGATTAACATCATGCAAG |  |
| Genomic Island V | BACI_c51040 | 51040-for | TTTCTTACGCCATTGTCGGG | 327 bp |
| (12.5 kb) |  | 51040-rev | GTACAGTAACCCCAGTAATAG |  |
|  | BACI_c51070 | 51070-for | TTGCTTATCGCTGCATCGAG | 354 bp |
|  |  | 51070-rev | TGTACGACTGAGTTGATAAGG |  |
| Genomic Island VI | BACI_c54520 | 54520-for | AATTGTTGCTCGAGAGGCAG | 604 bp |
| (12.5 kb) |  | 54520-rev | TTTTACAACCTCTTGATATGGC |  |
|  | BACI_c54560 | 54560-for | AATTTTAATCACTATACAGGAGC | 756 bp |
|  |  | 54560-rev | CTGCCCTTGTTCGTACAAAC |  |
| Plasmid pCI-14 | BACI_pBAslCI1400090 | 1400090-for | CCAATAATTGTGGGCACTCTG | 448 bp |
| (14.2 kb) |  | 1400090-rev | CATCTACACCTAAGAACTTGTTGC |  |
|  | BACI_pBAslCI1400180 | 1400190-for | TCTCGAAGAATGTGACAGCC | 445 bp |
|  | BACI_pBAslCI1400190 | 1400190-rev | ATCAATCATACGGTTCTAAGCC |  |
