# Supplementary material for: Bacillus cereus Biovar Anthracis Causing Anthrax in Sub-Saharan Africa—Chromosomal Monophyly and Broad Geographic Distribution
Source: PLoS Negl Trop Dis. 2016 Sep 8;10(9):e0004923. doi: 10.1371/journal.pntd.0004923 (PMC5015827; doi:10.1371/journal.pntd.0004923)
Supplement: S4 Table — (DOCX) [file pntd.0004923.s005.docx]

**S4 Table.** **Reciprocal Blast Average nucleotide identity Percentage using Jspecies.**

| Strains | CI | CAR (gor.) | CAR (ele.) | CAM | 14-0024-1 |
| --- | --- | --- | --- | --- | --- |
| Bacillus anthracis 3154 | 97.89/97.72 | 97.84/97.55 | 97.84/97.44 | 97.84/97.66 | 97.9/97.58 |
| Bacillus anthracis 3166 | 97.96/97.89 | 97.9/97.72 | 97.91/97.61 | 97.9/97.83 | 97.89/97.8 |
| Bacillus anthracis A0174 | 97.9/97.79 | 97.86/97.61 | 97.85/97.49 | 97.85/97.72 | 98.74/97.7 |
| Bacillus anthracis A0193 | 97.91/97.84 | 97.88/97.69 | 97.88/97.56 | 97.88/97.79 | 96.32/97.77 |
| Bacillus anthracis A0248 | 97.93/97.88 | 97.88/97.72 | 97.88/97.6 | 97.87/97.82 | 97.88/97.8 |
| Bacillus anthracis A0389 | 97.95/97.86 | 97.92/97.7 | 97.91/97.58 | 97.91/97.79 | 98.32/97.76 |
| Bacillus anthracis A0442 | 97.93/97.83 | 97.9/97.67 | 97.9/97.54 | 97.9/97.77 | 97.42/97.75 |
| Bacillus anthracis A0465 | 97.94/97.85 | 97.9/97.7 | 97.9/97.57 | 97.89/97.79 | 96.23/97.76 |
| Bacillus anthracis A0488 | 97.94/97.86 | 97.89/97.69 | 97.89/97.58 | 97.89/97.8 | 97.16/97.77 |
| Bacillus anthracis A1055 | 97.89/97.83 | 97.85/97.68 | 97.84/97.53 | 97.84/97.76 | 98.42/97.71 |
| Bacillus anthracis A2012 | 99.77/97.79 | 99.75/97.66 | 99.74/97.54 | 99.76/97.75 | 98.14/97.72 |
| Bacillus anthracis Ames Ancestor | 97.96/97.88 | 97.89/97.72 | 97.89/97.6 | 97.89/97.82 | 97.91/97.8 |
| Bacillus anthracis Australia 94 | 97.94/97.87 | 97.89/97.72 | 97.89/97.6 | 97.89/97.81 | 97.9/97.79 |
| Bacillus anthracis BF1 | 97.94/97.87 | 97.91/97.72 | 97.91/97.6 | 97.9/97.81 | 97.47/97.8 |
| Bacillus anthracis CDC 684 | 97.97/97.89 | 97.93/97.72 | 97.93/97.61 | 97.92/97.83 | 99.58/97.8 |
| Bacillus anthracis CNEVA 9066 | 97.97/97.88 | 97.94/97.72 | 97.94/97.61 | 97.93/97.82 | 97.92/97.8 |
| Bacillus anthracis Carbosap | 97.97/97.45 | 97.93/97.32 | 97.93/97.36 | 97.93/97.47 | 96.29/97.76 |
| Bacillus anthracis Gmb1 | 97.84/97.71 | 97.79/97.55 | 97.79/97.45 | 97.78/97.65 | 99.63/97.6 |
| Bacillus anthracis H9401 | 97.94/97.88 | 97.89/97.72 | 97.89/97.6 | 97.88/97.82 | 98.76/97.8 |
| Bacillus anthracis Heroin Ba4599 | 97.95/97.88 | 97.91/97.74 | 97.91/97.62 | 97.91/97.83 | 96.21/97.8 |
| Bacillus anthracis Kruger B | 97.92/97.85 | 97.88/97.7 | 97.88/97.58 | 97.88/97.79 | 97.88/97.78 |
| Bacillus anthracis Sen2Col2 | 97.84/97.71 | 97.79/97.55 | 97.79/97.44 | 97.79/97.65 | 99.64/97.6 |
| Bacillus anthracis Sen3 | 97.83/97.71 | 97.76/97.55 | 97.76/97.44 | 97.76/97.64 | 97.82/97.6 |
| Bacillus anthracis Tsiankovskii I | 97.87/97.71 | 97.71/97.81 | 97.59/97.59 | 97.81/97.87 | 97.92/97.79 |
| Bacillus anthracis UR 1 | 97.95/97.9 | 97.89/97.16 | 97.89/97.59 | 97.89/97.81 | 97.89/97.8 |
| Bacillus anthracis Vollum | 97.94/97.84 | 97.89/97.71 | 97.89/97.59 | 97.89/97.83 | 97.9/97.8 |
| Bacillus anthracis WNA USA6153 | 97.94/97.88 | 97.88/97.73 | 97.88/97.6 | 97.88/97.82 | 98.1/97.8 |
| Bacillus cereus 03BB102 | 97.28/97.26 | 97.27/97.04 | 97.27/96.94 | 97.26/97.21 | 97.3/97.19 |
| Bacillus cereus 03BB108 | 96.92/96.72 | 96.92/96.43 | 96.92/96.73 | 96.92/96.96 | 96.97/96.86 |
| Bacillus cereus AH820 | 97.53/97.58 | 97.55/97.42 | 97.54/97.29 | 97.55/97.53 | 99.25/97.45 |
| Bacillus cereus E33L | 96.89/97.09 | 96.87/96.93 | 96.87/96.82 | 96.86/97.01 | 96.9/97 |
| Bacillus cereus G9241 | 94.81/96.12 | 94.83/91.93 | 94.85/91.6 | 94.85/94.31 | 94.81/94.73 |
| Bacillus thuringiensis Al Hakam | 97.36/97.32 | 97.39/97.17 | 97.38/97.09 | 97.38/97.31 | 97.4/97.24 |
| Bacillus thuringiensis serovar konkukian 97 27 | 97.46/97.4 | 97.5/97.26 | 97.5/97.13 | 97.49/97.35 | 99.55/97.3 |
